# Harnessing Lactate Metabolism for Radiosensitization

**DOI:** 10.3389/fonc.2021.672339

**Published:** 2021-07-23

**Authors:** Kevin X. Liu, Emily Everdell, Sharmistha Pal, Daphne A. Haas-Kogan, Michael G. Milligan

**Affiliations:** ^1^ Department of Radiation Oncology, Brigham and Women’s Hospital, Dana-Farber Cancer Institute, Boston Children’s Hospital, Harvard Medical School, Boston, MA, United States; ^2^ Albany Medical College, Albany, NY, United States; ^3^ Department of Radiation Oncology, Dana-Farber Cancer Institute, Boston, MA, United States

**Keywords:** lactate metabolism, Warburg phenomenon, radiation therapy, radiosensitization, synergistic effects

## Abstract

Cancer cells rewire their metabolism to promote cell proliferation, invasion, and metastasis. Alterations in the lactate pathway have been characterized in diverse cancers, correlate with outcomes, and lead to many downstream effects, including decreasing oxidative stress, promoting an immunosuppressive tumor microenvironment, lipid synthesis, and building chemo- or radio-resistance. Radiotherapy is a key modality of treatment for many cancers and approximately 50% of patients with cancer will receive radiation for cure or palliation; thus, overcoming radio-resistance is important for improving outcomes. Growing research suggests that important molecular controls of the lactate pathway may serve as novel therapeutic targets and in particular, radiosensitizers. In this mini-review, we will provide an overview of lactate metabolism in cancer, discuss three important contributors to lactate metabolism (lactate dehydrogenase, monocarboxylate transporters, and mitochondrial pyruvate carrier), and present data that inhibition of these three pathways can lead to radiosensitization. Future research is needed to further understand critical regulators of lactate metabolism and explore clinical safety and efficacy of inhibitors of lactate dehydrogenase, monocarboxylate transporters, and mitochondrial pyruvate carrier alone and in combination with radiation.

## Introduction

At the most fundamental level, cells must be self-reliant—producing sufficient ATP and biosynthetic compounds to fuel their ongoing survival and proliferation (1). However, the ability of tumor cells to reprogram their metabolic activity in order to promote their own survival is one of the defining hallmarks of cancer (2). Dating all the way back to the 1920s and the pioneering work of Otto Warburg, lactate has long been identified as a major player in cancer metabolism (3). In his work, Dr. Warburg noted that many cancer cells uptake large amounts of glucose and preferentially produce lactate through glycolytic pathways, even in the presence of oxygen (4). This phenomenon has been observed across many different neoplasms and serves as the basis for tumor detection using glucose tracers with positron emission tomography (PET) (5–9). Recent studies have further shown that lactate plays a critical role in fueling tumor progression, remodeling the tumor microenvironment (TME), and inducing treatment resistance (10). Even more so, research has begun to reveal how lactate metabolism may be used to influence the radiosensitivity of tumors (11–13). This mini-review will provide a general overview of lactate metabolism and its role within diverse cancers, and specifically, summarize recent studies that suggest an interplay between lactate metabolism and response to radiation.

## Lactate Pathway

**Figure 1:**
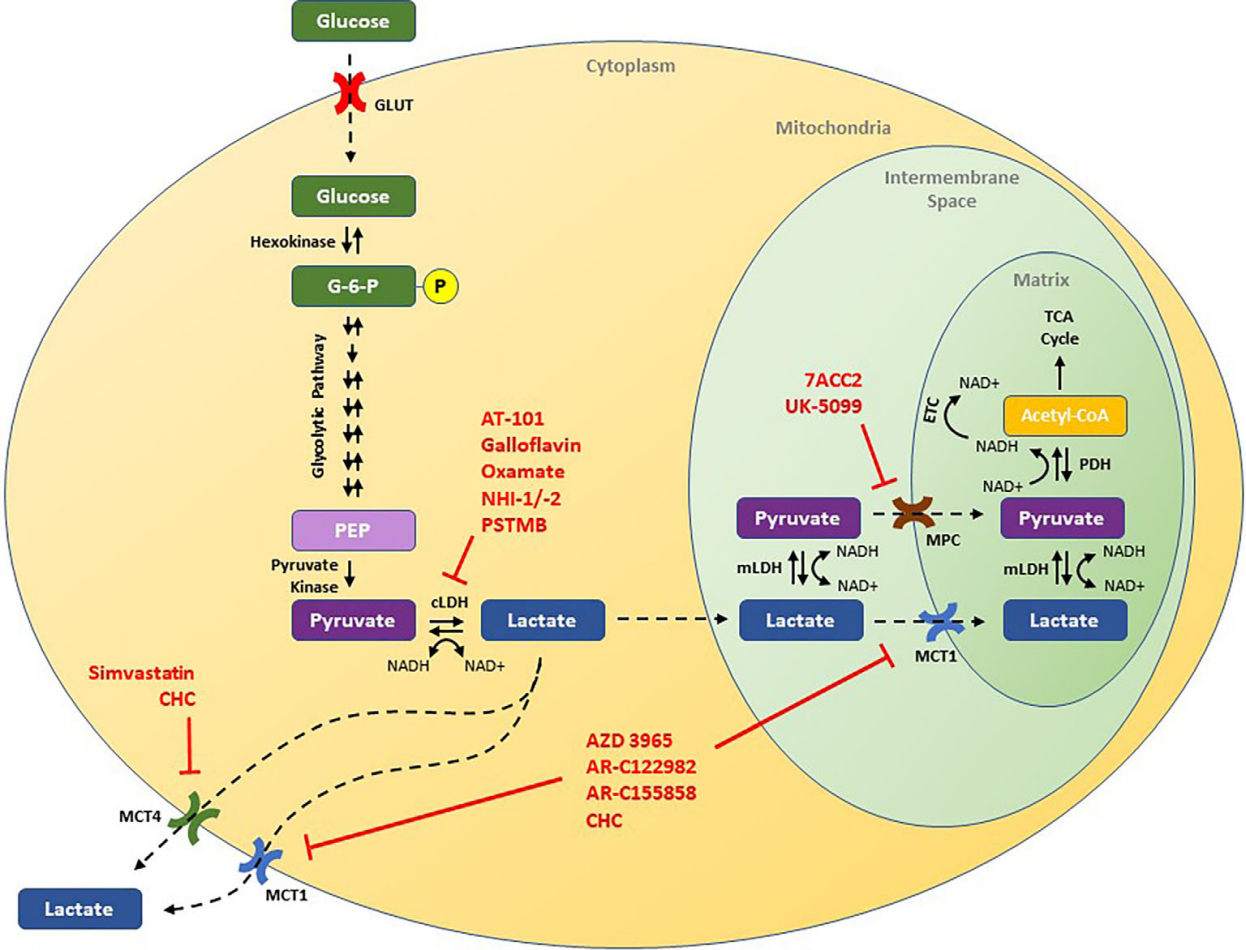
Schematic of the lactate pathway and illustration of novel therapeutic strategies that have been shown to decrease tumor growth in preclinical studies. Drugs discussed in this review are presented in red and are shown by their putative target of action. ECT, electron transport chain; G-6-P, glucose-6-phosphate; GLUT, glucose transporter; cLDH, cytosolic lactate dehydrogenase; mLDH, mitochondrial lactate dehydrogenase; MCT1, monocarboxylate transporter 1; MCT4, monocarboxylate transporter 4; MPC, mitochondrial pyruvate carrier; NAD+, nicotinamide adenine dinucleotide (oxidized); NADH, nicotinamide adenine dinucleotide (reduced); PEP, phosphoenolpyruvate; PDH, pyruvate dehydrogenase.

In normal human cellular physiology, glucose serves as a major source of lactate production (**Figure 1**). Glucose is most commonly taken into cells *via* facilitated diffusions through glucose transporter proteins (GLUT) (14). Once inside, glucose is phosphorylated to glucose-6-phosphate by hexokinase, effectively entrapping it in the cell (15). In the cytoplasm, glucose-6-phosphate is routed through several oxygen-independent glycolytic reactions to generate two ATP and two molecules of pyruvate, among other products (15). Under normal aerobic conditions, the majority of this pyruvate is then transported into the mitochondria *via* either mitochondrial pyruvate carrier (MPC) or after conversion to lactate *via* monocarboxylate transporters (MCTs), and undergoes oxidative phosphorylation, generating another 32 to 34 ATP per glucose molecule *via* the tricarboxylic acid cycle and electron transport chain (16, 17). However, under anaerobic conditions, cells are unable to rely on oxidative phosphorylation to balance their redox state, and pyruvate is preferentially converted to lactic acid through an enzymatic reaction catalyzed by cytosolic lactate dehydrogenase (LDH) (18). Within cells, lactic acid almost completely dissociates to lactate and H^+^, and lactic acid accumulation leads to acidification of the cytoplasm and potent inhibition of further glycolysis (18, 19). As such, proper physiological functioning depends on the efflux of lactate out of the cell, and transport across mitochondrial and cellular membranes requires MCTs. Once outside of a glycolytic cell, the excreted lactate is ultimately destined for one of several possible fates. Under physiological conditions, tissues like the heart, brain, and skeletal muscles can use lactate as a fuel source, while the liver can convert circulating lactate into glucose through the Cori cycle (20, 21). Indeed, a growing body of literature has shown that such “shuttling of lactate” between organs plays an important role in the overall regulation of metabolism (22).

## Lactate and Cancer

In blood and healthy tissues, the physiological concentration of lactate is roughly 1.0 to 3.0 mmol/L. However, in cancer cells, lactate concentrations may be up to an order of magnitude higher. Such high concentrations of lactate have been shown to arise primarily from enhanced rates of glycolysis (23). Interestingly, despite its inherent inefficiency in terms of ATP production, high rates of glycolysis have been observed in many cancer cells, even under fully aerobic conditions and with intact oxidative phosphorylation function (23). There are two major theories regarding the preferential dependence of cancer cells on glycolysis. First, the rate of ATP production through glycolysis is much more rapid than oxidative phosphorylation allowing cells to meet changing energy requirements, and second, glycolysis produces many intermediate biosynthetic molecules required by rapidly proliferating cells (24, 25). Regardless of teleology, there are a number of adaptive enzymatic alterations that lead to this so-called, “Warburg phenotype,” including changes in the function of hexokinase 2 (HK2), pyruvate kinase type M2 (PKM2), GLUT1, LDH, MCTs, and pyruvate dehydrogenase (PDH) (26–32). The resulting high concentrations of lactate have been further implicated in a wide range of tumoral aberrations, including changes in the TME, immune suppression, and metastasis—where increasing tumoral lactate concentrations are associated with an increased risk of metastatic dissemination (10, 33–39).

Radiotherapy is a curative treatment modality in diverse cancer types, including breast cancer (40, 41), head and neck cancers (42), brain cancer (43), and many pediatric solid tumors (44–46). Furthermore, radiotherapy remains an essential option for palliation such that approximately 50% of patients with cancer will receive radiation treatments during their disease course (47, 48). Recent studies have found that high rates of glycolysis and the build-up of lactate likely contribute to radioresistance in many tumor types through diverse mechanisms including antioxidant protective effects and promoting an immunosuppressive TME (39, 49–53). Interestingly, through impacts on several cellular processes including LDH and PDH, radiation itself can also promote lactate production, which, in turn, may drive a degree of radioresistance (39, 54–56). Within the extensive cellular machinery involved with lactate metabolism, three promising targets, LDH, MCT1/4, and MPC, have been shown to modulate radiosensitivity.

## Lactate Dehydrogenase (LDH)

LDH is a nicotinamide adenine dinucleotide (NAD+) oxidoreductase enzyme that catalyzes the conversion between pyruvate and lactate (57). While constitutively active in aerobic conditions, LDH expression is upregulated in hypoxic environments *via* HIF-1*α* (58). LDH is a tetrameric enzyme comprised of 2 subunits that can combine in any of 5 combinations. The most common subtype, known as LDHA, preferentially reduces pyruvate to lactate, and is frequently over-expressed in many tumors (10). In addition, LDHA has been shown to catalyze a number of “non-canonical” reactions, including the formation of an “onco-metabolite”—2-hydroxyglutarate—in acidic and anaerobic environments, thus promoting oncogenesis (59–65). Recent studies demonstrate that 2-hydroxyglutarate can promote a transcriptional program of genes that regulate proliferation and growth by inhibiting histone demethylation and TET-mediated DNA demethylation (59–65). Clinically, increased tumoral LDHA expression is associated with poorer clinical outcomes and recent studies suggest it may serve as a prognostic biomarker (66–68).

Knockdown studies of both PKM2 and LDHA have been shown to reduce ATP production, inhibit cell growth, decrease invasiveness, and induce oxidative stress and radiosensitivity in cancer cells (39, 69, 70). Several clinical and pre-clinical studies have further analyzed the effects of both selective and non-selective inhibitors of LDHA on cancer cells. AT-101, a naturally occurring compound derived from cottonseed, is an oral non-selective inhibitor of LDH that additionally inhibits the anti-apoptotic proteins Bcl-2, Bcl-xL, Bcl-W, and Mcl-1 while simultaneously stimulating pro-apoptotic signaling (71, 72). A study of AT-101 monotherapy in 23 men with metastatic castrate-resistant prostate cancer showed that a dose of 20 mg/day was well tolerated and led to a >50% decrease in PSA in roughly 9% of patients (71). Heist and colleagues found that while AT-101 administered concurrently with topotecan was safe for patients with small cell lung cancer (SCLC) who had failed prior platinum-based chemotherapy, this regimen failed to show significant activity with only 8% of patients experiencing a partial response (73). Similarly, Baggstrom et al. failed to demonstrate efficacy in patients with chemo-sensitive recurrent SCLC (74). However, in pre-clinical studies, FX11—a derivative of AT-101 that selectively inhibits LDHA over LDHB—effectively inhibited tumorigenesis *in vivo* using human lymphoma and pancreatic tumor xenograft models (38). When combined with a small molecule inhibitor of NAD+ synthesis, FX11 was further able to induce tumor regression in the lymphoma xenograft model (38). Another study found that both galloflavin, a polyphenol inhibitor of LDH, and oxamate, a competitive analogue of pyruvate, disrupted the heat shock response in cultured hepatocellular carcinoma (HCC) cells and induced cellular senescence (75). Furthermore, two recent studies found that inhibition of LDHA in glioblastoma cell lines with either oxamate or the selective inhibitors, NHI-1 and NHI-2, improved chemotherapy and radiation sensitivity and triggered apoptosis and differentiation of cancer stem cells (76, 77). More recently, PSTMB—a novel allosteric inhibitor of LDH—was found to reduce cellular proliferation in *in vitro* models of lung cancer, breast cancer, melanoma, HCC, and colon cancer (78). In cultured colon cancer cells, PSTMB reduced LDH activity in both aerobic and anaerobic conditions without altering LDH expression, and increased reactive oxygen species (ROS) formation (78).

Despite clinical and pre-clinical interest in LDH inhibitors, there have been few studies of these agents in combination with radiotherapy. Koukourakis, et al. assessed the effects of LDH blockade on the treatment sensitivity of 2 glioblastoma cell lines, U87MG and the more radio-resistant T98G. Silencing LDHA gene expression or inhibiting LDH with oxamate led to enhanced sensitivity to both radiation and temozolomide, with more pronounced effects observed in the T98G cell line (76). Another study by Zhai and colleagues showed that oxamate increased radiation sensitivity primarily by enhancing mitochondrial ROS generation, which in turn promoted apoptosis in two nasopharyngeal cancer cell lines (79). Yang et al. found that radiotherapy increased lactate concentrations in the TME which then led to localized immunosuppression *via* MDSCs in murine models with explanted human pancreatic cancer cells, and administration of the selective LDHA inhibitor GSK2837808A concurrently with radiation improved antitumoral T-cell response and reduced tumor progression (39). These results suggest that lactate is at least partially responsible for the observed radiotherapy-induced immunosuppression. In a different tact, Judge et al. observed that high lactate concentrations activated latent TGF-β, leading to excessive fibrosis and found that increased LDHA expression correlated with higher rates of pulmonary fibrosis in patients treated with radiotherapy (80). Treatment with AT-101 four weeks after exposing C57BL/6 mice to total-body and thoracic radiation showed significantly decreased TGF-β expression and rates of pulmonary fibrosis (80). Two early phase clinical trials are examining the safety of concurrent chemoradiation with AT-101 in glioblastoma and esophageal and esophagogastric junction cancers (**Table 1**). Overall, these results highlight many of the potential advantages of LDH inhibitors in combination with radiotherapy; however, significant work still remains in order to determine clinical utility.

**Table 1:** Preclinical studies and clinical trials exploring lactate pathway targets with radiotherapy in cancer.

Preclinical Studies				
Target	Inhibitors	*In vitro/In vivo* model	Results	Reference
LDH	Oxamate	*In vitro:* U87MG and T98G glioblastoma cell lines	Oxamate and radiation decreased RD50	(76)
LDH	Oxamate	*In vitro:* CNE-1 and CNE-2 nasopharyngeal carcinoma cell lines	Oxamate and radiation increased apoptosis at 24 hours after radiation and increased radiation-induced inhibition of clonogenic survival. Oxamate and radiation decreased tumor growth in *vivo*	(79)
		*In vivo:* CNE-1 xenograft tumors		
LDH	GSK2837808A	*In vivo:* Panc-02-luciferase orthotopic tumors	GSK2837808A and radiation decreased tumor growth and MDSC activation, and increased cytotoxic CD8+ T cells within the tumor in *vivo*	(39)
MCT	AR-C122982, AR-C155858, simvastatin, 2-cyano-3-(4-hydroxyphenyl)-2-propenoic acid (CHC)	*In vitro:* CAL27 oral squamous cell carcinoma cell line	AR-C122982, simvastatin, or CHC and radiation decreased cell proliferation	(81)
MCT	AZD3965	*In vitro:* H526 small cell lung cancer cell line	AZD3965 and radiation increased intracellular lactate concentration, and decreased tumor growth and improved survival in *vivo*	(82)
		*In vivo:* H526 small cell lung cancer xenograft tumors		
MPC	7-aminocarboxycoumarin 2 (7ACC2), UK-5099	*In vivo:* SiHa cervical cancer xenograft tumors	7ACC2 or UK-5099 and radiation decreased tumor growth	(11)
**Clinical studies**				
**Target**	**Treatment regimen**	**Diagnosis**	**Results**	**Reference**
LDH	AT-101 and chemoradiation with docetaxel and 5-fluorouracil (NCT00561197)	Locally advanced esophageal or gastroesophageal junction cancer	Ongoing trial	
LDH	AT-101 and chemoradiation with temozolomide or temozolomide alone (NCT00390403)	Glioblastoma Multiforme	Ongoing trial	

MDSC, myeloid-derived suppressor cells; RD50, radiation dose resulting in 50% cell viability.

## Monocarboxylate Transporters

MCTs constitute 14 isoforms of membrane transport proteins that aid in the absorption and efflux of a wide range of biological compounds (83). Ubiquitously expressed, MCT1 primarily mediates import of lactate along with other monocarboxylates (84–87). On the other hand, MCT4 expression is regulated by hypoxia through a HIF-1*α*-dependent mechanism. MCT4 has lower affinity for lactate compared to MCT1, and predominantly participates in lactate efflux (84–87). MCT1 and MCT4 are overexpressed in many cancer types, and their upregulation correlates with worse overall prognosis (17, 88–91). MCT1 and MCT4 may help not only maintain metabolic balance for oxidative and glycolytic cancer cells, respectively, but may also promote an immunosuppressive milieu by increasing the acidity of the TME secondary to the accumulation of lactate (92, 93). Increased acidity has been found to decrease CD8+ T-cell cytotoxicity and CD8+ T-cell-mediated cytokine release; and induce macrophage polarization to an immunosuppressive M2 state (3, 94). Thus, studies have investigated MCTs as a therapeutic target and in particular, a radiosensitizer, in various cancers (81, 82, 89, 95–98).

Numerous studies have found that knockdown or inhibition of MCT1 and/or MCT4 decreases lactate levels and tumor cell growth, migration, and invasion *in vitro* and *in vivo* for diverse cancers, including bladder cancer, breast cancer, colon cancer, glioblastoma, and liver cancer (89, 95–100). Interestingly, in a model of Burkitt’s lymphoma, a selective small molecule inhibitor of MCT1, AZD3965, also decreased lipid biosynthesis after lactate build-up, and specifically, levels of phosphocholine were significantly decreased by inhibition of choline kinase α expression and *de novo* phosphocholine synthesis. Furthermore, in the TME, AZD3965-treated tumors also displayed greater interaction with dendritic cells—increasing tumor antigen presentation—and natural killer cells—leading to direct killing of tumor cells (101).

Two studies have examined the combination of MCT inhibition and radiation in tumor models. Brandstetter et al. studied the effects of multiple MCT inhibitors with or without radiation on the oral squamous cell carcinoma (SCC) cell line CAL27 *in vitro*. Specifically, they analyzed MCT1 inhibitors, AR-C122982 and AR-C155858, the MCT4 inhibitor, simvastatin, and the non-specific MCT inhibitor, 2-cyano-3-(4-hydroxyphenyl)-2-propenoic acid (CHC) (81). Though the MCT inhibitors differed in their potency and efficacy, treatment decreased cell proliferation, viability, and wound healing (81). The combination of radiation with specific MCT inhibitors further resulted in enhanced anti-proliferative activity (81). Currently, it is not known whether these other pathways may further contribute to the effects seen in this study.

Many studies have found that AZD3965, a selective small molecule inhibitor of MCT1, is effective in inhibiting tumor growth in many different preclinical models of cancer (82, 99–104). Two studies demonstrated that AZD3965 inhibited bidirectional lactate transport leading to both accumulation of intracellular lactate (with greater effects observed in hypoxic conditions), and antitumor activity in SCLC models *in vitro* and in xenograft models (82, 99). Bola et al. found that radiation alone did not affect intracellular lactate in H526 SCLC cells, but when delivered in combination with AZD3965, intracellular lactate concentration significantly increased (82). Furthermore, compared with radiation alone, AZD3965 for seven days with concurrent radiation delivered on days 3-5 significantly decreased tumor growth and improved survival with one mouse showing no tumor recurrence (82). Unfortunately, MCT1 inhibitors are ineffective in tumor cells that highly express MCT4, which suggests that MCT4 expression may serve as a biomarker for patient selection and predictor of response to anti-MCT1 therapy (98, 99).

Of the MCT1 inhibitors, AZD3965 has entered early phase clinical trials. Preliminary results from a phase I study investigating the safety of AZD3965 in patients with refractory advanced solid malignancies found that AZD3965 was well tolerated. While the most common side effects were nausea and fatigue, patients also experienced expected on-target effects of retinal electroretinographic changes that were dose-limiting at 20 mg daily and increased urinary ketones (105). One patient had exacerbation of previously undiagnosed tumor-associated lactic acidosis, which was dose-limiting (105). Future research is still needed to explore the tolerability and efficacy of AZD3965 and other MCT inhibitors alone and in combination with radiation.

## Mitochondrial Pyruvate Carrier

MPC is formed by two proteins encoded by genes *MPC1* and *MPC2*. It transports pyruvate from the cytoplasm into mitochondria, and sits at the crossroads of glycolysis, mitochondrial oxidative phosphorylation, and lactate production (106, 107). In highly glycolytic tumors, decreased MPC expression can lead to aerobic glycolysis and shunting to glutaminolysis, ultimately leading to greater tumor proliferation. On the other hand, tumors that are more dependent on oxidative phosphorylation may be more sensitive to alterations in MPC-mediated pyruvate transport (106). Recent studies have also found that lactate accumulation in tumors can promote the synthesis of intermediates of the tricarboxylic acid cycle, further supporting cell proliferation (12, 108, 109). Thus, there is growing interest in MPC as a regulator of both oxidative phosphorylation and lactate production in tumorigenesis.

Recently, 7-aminocarboxycoumarin 2 (7ACC2) was identified as a novel MPC inhibitor that led to downstream reductions in lactate influx and delays in tumor growth within *in vitro* models of cervical cancer, colorectal cancer, breast cancer, hypopharyngeal SCC, and pancreatic cancer (11, 110–112). Corbet et al. found that 7ACC2 blocked MPC activity, thereby inhibiting pyruvate metabolism and subsequently blocking lactate influx consistent with another known MPC inhibitor, UK-5099 (11). In a spheroid model using FaDu hypopharyngeal SCC cells, treatment with MPC inhibitors produced cytotoxic effects and led to decreased hypoxia in the spheroids (11). In SiHa cervical cancer xenograft models, the combination of 7AAC2 with radiation using either 16 Gy in one fraction or 20 Gy in five fractions, led to significantly decreased tumor growth compared with 7AAC2 or radiation monotherapy. Similar results were also observed *in vivo* using shRNA targeting MPC1 or UK-5099 (11). These preclinical data suggest that MPC represents a novel target warranting further clinical investigation both alone and in combination with radiation.

## Future Directions

Many preclinical studies have identified multiple targets within the lactate metabolic pathway that play a role in radiosensitization, and future research is ongoing to identify novel targets for lactate metabolism. Studies exploring safety of these targets are still needed, particularly for patients at risk for metabolic acidosis either from co-morbidities or prior cancer therapy. Furthermore, it remains important to note that different solid tumors may have unique alterations in lactate metabolism and intratumoral metabolic heterogeneity may also cause differential response to inhibition of lactate metabolism (10, 108, 113, 114). These characteristics are important considerations for future studies and increasingly support identifying tumor types in which harnessing radiosensitizing properties through lactate metabolism inhibition has the greatest therapeutic benefit. For example, additional imaging techniques, such as ^13^C magnetic resonance spectroscopy, can better provide dynamic imaging of lactate metabolic reprogramming (115–117). A recent clinical trial explored de-escalation of radiation to 30 Gy for patients with human papillomavirus-associated oropharyngeal tumors who had no hypoxia at baseline using dynamic fluorine-18-labeled fluoromisonidazole PET or resolution of hypoxia during intratreatment PET; while patients with persistent hypoxia received 70 Gy (118). Identifying patients with hypoxic tumors or tumors with specific alterations of lactate metabolism may allow for improved patient selection for future clinical trials involving radiation and inhibitors of lactate metabolic pathways.

## Conclusion

A growing body of evidence has shown that, in addition to its use as a fuel source, lactate also promotes tumor growth. Interestingly, elevated lactate levels and lactate-mediated downstream pathways can cause changes in transcriptional programming (59–65), tumor immune microenvironment (10, 39), lipid synthesis (101), among others (3). The effects of these downstream changes, particularly with regards to decreasing the levels of ROS, can contribute to radio-resistance (3). Recent studies have found that inhibitors of LDH, MCT, and MPC can serve as radiosensitizers in models of glioblastoma, pancreatic cancer, SCLC and cervical cancer (11, 39, 76, 82). There remains limited clinical investigation of these inhibitors with radiation as only two early phase clinical trials are studying AT-101 in combination with radiation (**Table 1**). Future research is needed to understand the mechanisms by which regulators of lactate metabolism promote tumorigenesis, identify tumor subtypes that are uniquely dependent on lactate pathways, and to further explore targeted inhibitors of this pathway in preclinical and clinical studies.

## Author Contributions

KL, SP, DH-K, and MM contributed to the conception and design of this study. KL, EE, and MM wrote the first draft of the manuscript. All authors contributed to the article and approved the submitted version.

## Conflict of Interest

The authors declare that the research was conducted in the absence of any commercial or financial relationships that could be construed as a potential conflict of interest.

## Publisher’s Note

All claims expressed in this article are solely those of the authors and do not necessarily represent those of their affiliated organizations, or those of the publisher, the editors and the reviewers. Any product that may be evaluated in this article, or claim that may be made by its manufacturer, is not guaranteed or endorsed by the publisher.
